# High Intensity Interval- vs Resistance or Combined- Training for Improving Cardiometabolic Health in Overweight Adults (Cardiometabolic HIIT-RT Study): study protocol for a randomised controlled trial

**DOI:** 10.1186/s13063-016-1422-1

**Published:** 2016-06-24

**Authors:** Robinson Ramírez-Vélez, Alejandra Hernandez, Karem Castro, Alejandra Tordecilla-Sanders, Katherine González-Ruíz, Jorge Enrique Correa-Bautista, Mikel Izquierdo, Antonio García-Hermoso

**Affiliations:** Centro de Estudios en Medición de la Actividad Física (CEMA), Escuela de Medicina y Ciencias de la Salud, Universidad del Rosario, Cra. 24 No. 63C-69, Bogotá, D.C Colombia; Grupo de Ejercicio Físico y Deportes, Facultad de Salud, Programa de Fisioterapia, Universidad Manuela Beltrán, Bogotá, D.C Colombia; Department of Health Sciences, Public University of Navarra, Pamplona, Navarra Spain; Laboratorio de Ciencias de la Actividad Física, el Deporte y la Salud, Universidad de Santiago de Chile, USACH, Santiago, Chile

**Keywords:** Exercise, Risk factor, Cardiovascular disease, Overweight

## Abstract

**Background:**

Although evidence shows the positive health effects of physical activity, most of the adult population in Colombia are sedentary. It is, therefore, important to implement strategies that generate changes in lifestyle behaviours. This protocol describes a study in which we will compare the effects of 12 weeks of high-intensity interval training (HIIT), resistance training (RT) or combined training (HIIT + RT) on the improvement of body composition, endothelial function, blood pressure, blood lipids, and cardiorespiratory fitness in a cohort of sedentary, overweight adults (aged 30–50 years).

**Methods/design:**

Sixty sedentary, overweight adults attending primary care in Bogotá, Colombia will be included in a factorial randomised controlled trial. Participants will be randomly assigned to the following intervention groups: (1) non-exercise group: usual care with dietary support, (2) HIIT group: 4 × 4-min intervals at 85–95 % maximum heart rate (HRmax) (with the target zone maintained for at least 2 minutes), interspersed with a 4-min recovery period, at 65 % HRmax, (3) RT group: completing a resistance circuit (including upper and lower muscle groups) as many times as needed according to subject’s weight until an expenditure of 500 kcal at 40–80 % of one-rep max (1RM) has been achieved, and (4) combined group: HIIT + RT. The primary end point for effectiveness is vascular function as measured by flow-mediated vasodilatation 1 week after the end of exercise training.

**Discussion:**

The results of this study will provide new information about the possible effect of the programme in improving the cardiometabolic health of overweight adults, making a more efficient use of an adult’s resources over time.

**Trial registration:**

ClinicalTrials.gov ID: NCT02715063. Registered on 8 March 2016.

**Electronic supplementary material:**

The online version of this article (doi:10.1186/s13063-016-1422-1) contains supplementary material, which is available to authorized users.

## Background

The prevalence of obesity has increased worldwide [1] among both children and adults, and obesity is associated with an increased risk for cardiovascular diseases (CVD) [2, 3]. Substantial evidence indicates that increased body weight and body fat distribution are associated with a higher frequency of adverse health consequences, including hypertension, CVD, metabolic disorders, osteoarthritis, gallbladder stone disease, asthma and multiple malignancies [4, 5]. International organisations [6, 7] and previous epidemiological cross-sectional studies have suggested that individuals with a large accumulation of body fat in the abdominal region are at greater risk for the development of metabolic syndrome [8, 9]. In addition to obesity, hypertension, dyslipidaemia and low cardiorespiratory fitness (CRF) are also modifiable risk factors associated with the risk for CVD [10, 11]. Furthermore, it has become increasingly clear that low CRF may exacerbate the risk of CVD mortality, and that increasing peak CRF to higher than 5 peak metabolic equivalents (MET) can reduce and perhaps eliminate the mortality rate associated with dyslipidaemia, obesity, type 2 diabetes mellitus and hypertension [12].

The health benefits of exercise training are well documented; it is necessary for healthy growth and development because it optimises cardiometabolic function and prevents chronic disease [13, 14]. Additionally, its benefits are not only biological, but also include psychosocial advantages [15]. The most recent guidelines promoted by the World Health Organisation (WHO) recommend a minimum of 150 min of moderate-intensity physical activity (3 to <6 MET) or 75 min of vigorous-intensity physical activity (≥6 MET per week or any equivalent combination for health benefits, and 300 min of moderate-intensity physical activity or 150 min of vigorous-intensity physical activity per week for additional health benefits [16, 17].

Previous studies have reported improvement in endothelial function in different disorders, such as obesity, diabetes mellitus and metabolic syndrome, by increasing the production and bioactivity of nitric oxide [18–21]. In addition, a growing body of evidence has demonstrated comparable or superior improvements in cardiometabolic health outcomes using low-volume, high-intensity interval training (HIIT) compared to traditional moderate-intensity continuous training (MICT) [18, 19]. Other studies have revealed a strong relationship between vascular function and CRF [20, 21]. Thus, because HIIT is a potent method of improving CRF, several systematic and narrative reviews that have investigated the impact of HIIT relative to MICT on vascular function in clinical patients have emerged over recent years [18, 19, 22]. HIIT provides rapid physiological adaptations, as indicated by improvements in maximal oxygen uptake (VO_2_max), anaerobic threshold and stroke volume [23, 24]. Instead, it was suggested that the ability of HIIT to restore vascular homeostasis through enhancement in shear stress-induced nitric oxide bioavailability may be another important mechanism that explains the protective role of exercise against CVD development [25].

Interestingly, despite the prevalence of obesity and the existing multiple position stands promoting exercise for the treatment of obesity, there are few randomised trials that have directly compared the effects of sustained resistance training (RT), HIIT, or a combination of the two (RT + HIIT) to be as effective or more effective for improving cardiometabolic health in adults [26, 27]. Most of the published studies addressing RT and fat mass changes have compared RT to an inactive control group and not to HIIT. Furthermore, existing studies have not directly studied comparable amounts of HIIT and RT. A recent randomised controlled trial (RCT) suggests that adding plyometric exercises to a HIIT programme may be more beneficial than HIIT alone in young obese women [28]. Given the increasing burden of chronic disease, more research is needed to better understand the effect of different exercise modalities on these risk factors [29].

Thus, this paper describes the rationale, design, and methodologies used in a factorial randomised controlled trial (Cardiometabolic HIIT-RT Study), wherein we hypothesised that HIIT and combined training would result in greater improvements in vascular function compared to RT and usual clinical care.

## Methods/design

### Study design and setting

The present study is a RCT (ClinicalTrials.gov ID: NCT02715063). The Cardiometabolic HIIT-RT Study is a single-blind, randomised controlled, 2 × 2 factorial trial. The study received ethical approval from the Medical Research Ethics Committee of The University of Manuela Beltran (ID 06-1006-2014). Random allocation to treatment will be performed at the individual level.

### Procedures

#### Participants

Participants aged 30–50 years, who are sedentary (no participation in exercise more than once a week for the previous 6 months) with abdominal obesity: waist circumference (WC) at least 90 cm for men, and at least 80 cm for women, or with excess weight: body mass index (BMI) at least 25 kg/m^2^ for men and 35 kg/m^2^ for women, and who are identified as being willing and with almost immediate availability, will be enrolled. Eligible subjects for the present study and those interested in participating will be invited to a pre-test that includes an interview in a private health care institution (Clinica Rangel Pereira IPS) and further assessments performed at the Centre of Studies in Physical Activity Measurements (in Spanish, CEMA), School of Medicine and Health Sciences, University of Rosario, Bogotá, Colombia. Risks will be minimised by ruling out contraindications to the testing and training protocols via a health history and thorough physical examination prior to the testing sessions. Inclusion and exclusion criteria are provided in Table 1.

**Table 1:** Inclusion/exclusion criteria

Inclusion criteria	Exclusion criteria
Central obesity: waist circumference ≥90 cm (men); ≥80 cm (women), or with excess weight: body mass index ≥25 kg/m^2^ (men) and ≤ 35 kg/m^2^ (women)	Systemic infections
Meets at least one criteria for metabolic syndrome (IDF 2006): triglycerides ≥150 mg/dl; HDLc <40 mg/dL (men); <50 mg/dL (women); high blood pressure ≥130/85 mmHg and/or fasting plasma glucose ≥100 mg/dL	Weight loss or gain of >10 % of body weight in the past 6 months for any reason
Written informed consent	Currently taking medication that suppresses or stimulates appetite
Interested in improving cardiovascular health and physical fitness	Uncontrolled hypertension: systolic blood pressure 160 mmHg or diastolic blood pressure 95 mmHg on treatment
	Gastrointestinal disease, including self-reported chronic hepatitis or cirrhosis, any episode of alcoholic hepatitis or alcoholic pancreatitis within the past year, inflammatory bowel disease requiring treatment within the past year, recent or abdominal surgery (e.g. gastrostomy)
	Asthma
	Diagnosed diabetes (type 1 or 2), fasting impaired glucose tolerance (blood glucose 118 mg/dL), or use of any antidiabetic medications
	Currently taking antidepressant, steroid, or thyroid medication, unless dosage unstable (no change for 6 months)
	Current exerciser (>30 min organised exercise per week).
	Indication of unsuitability of current health for exercise protocol (Physical Activity Readiness Questionnaire, PARQ)
	Any other conditions which, in the opinion of the investigators, would adversely affect the conduct of the trial

#### Recruitment

Consecutive men or women with abdominal obesity or excess weight will be recruited from a private health care institution (Clinica Rangel Pereira, IPS) and one primary care institution (Universidad del Rosario, IPS) that receives referrals from both medical consultants in secondary care and primary care general practitioners in the capital district of Bogotá, Cundinamarca Department in the Andean region. This region is located at approximately 4°35′56″N 74°04′51″W and at an elevation of approximately 2625 m (min: 2500 m; max: 3250 m) above sea level [30]. Subjects who are interested in participating will be approached with further information and screened for pre-participation exercise habits using a cardiovascular and musculoskeletal checklist (i.e. the patient’s medical history, disease history, physical fitness, and more). A member of the research team will follow up with a phone call to screen for eligibility and explain the main requirements of the study. All participants will provide written informed consent before entering the study.

#### Blinding and randomisation methods

**Fig. 1:**
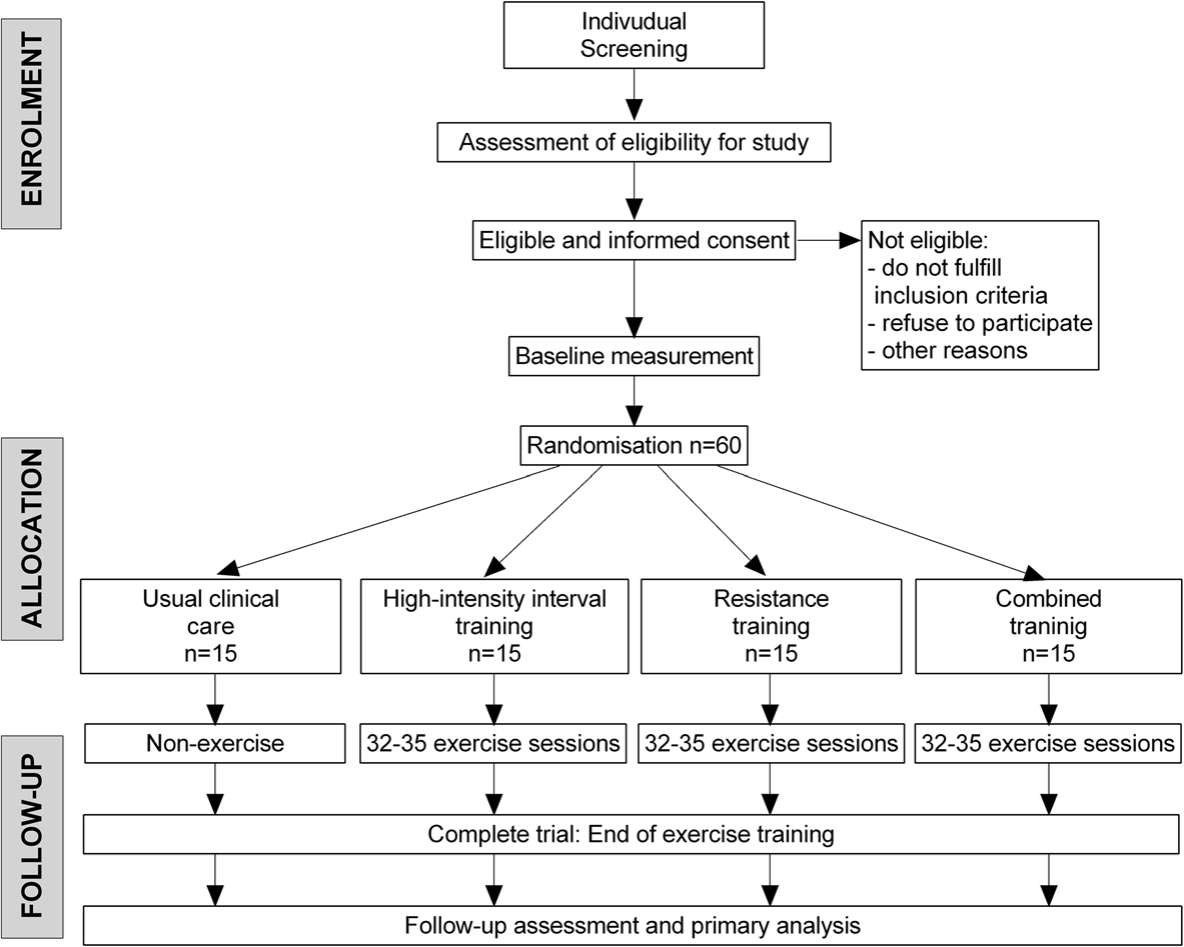
Consolidated Standards of Reporting Trial (CONSORT) guidelines flow diagram for enrolment and randomisation in the Cardiometabolic HIIT-RT Study

The randomisation into the four study arms will be performed by the Centre of Studies in Physical Activity Measurements at University of Rosario, Bogotá, Colombia, using block randomisation with a block size of four. Eligible participants will be randomly assigned after completing the baseline measurements to either the control or exercise training groups. The principal investigator will coordinate the allocation sequence, and randomisation will be computer-generated. All participants and study personnel (including investigators, trainers, and statisticians) will be blinded to treatment allocation throughout the trial protocol. Access to the allocation code will be restricted to one study statistician who will not perform the final study analyses. Randomisation will be conducted independently using sealed opaque envelopes. These procedures are also detailed in the study operations manual. Moreover, the importance of maintaining the blinding and allocation concealment will be reinforced by regularly scheduled conference calls at the sites and daily meetings with the field investigators (Fig. 1).

#### Intervention

The participants who are randomly assigned to the intervention group will participate in the cardiometabolic programme.

1. Usual clinical care group

This group will receive usual clinical care according to the consensus recommendations of the national goals for cardiovascular health promotion and disease reduction of the American Heart Association [31] and Colombian guidelines COLDEPORTES (in Spanish, Departamento Administrativo del Deporte, la Recreacion, la Actividad Fisica y el Aprovechamiento del Tiempo Libre) [32]. Participants will receive counselling about goals for cardiovascular health, as well as monitoring cardiovascular health over time in the Colombian population, key signs and symptoms, diet and screening for cardiometabolic risk factors. This group will be asked to maintain their level of activity during the 12-week study period.

2. High-intensity interval training (HIIT) group

The HIIT protocol will be completed with fast walking or running on a treadmill with the deck inclined to reach the desired intensity. We will calculate the training energy expenditure for participants’ age ranges associated with meeting the consensus public health recommendations from the WHO [16] and the US Department of Health and Human Services [17]. Each preparatory period starts with an exercise dose of 6 kcal kg^-1^ week^-1^, which will increase progressively by 2 kcal kg^-1^ week^-1^ until week 4, where it will remain at 12 kcal kg^-1^ week^-1^ for weeks 5 to 12.

*Preparatory training phase: weeks 1–4*

To initiate our study we will use a 4-week preparatory phase of training to bring all participants up their 300-kcal goal for the session. To accomplish this they will warm up at 65 % of maximum heart rate (HRmax) (5 min); exercise for 4 × 4-min intervals at 60–80 % HRmax, interspersed with a 4-min recovery period at 55 % HRmax, at a frequency of three times per week.

*Protocol of interval training: weeks 5–12*

**Fig. 2:**
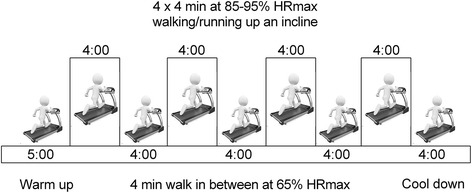
Example of a 30–40-min 4 × 4 high-intensity interval training (HIIT) session

The overall goal for the HIIT group is to perform exercise sessions in 4 × 4-min intervals at 85–95 % HRmax (with the target zone maintained for at least 2 minutes), interspersed with a 4-min recovery period at 65 % HRmax. During each exercise session, participants will adhere to the 12-kcal kg^-1^ week^-1^ energy expenditure format, equivalent to 500 kcal of expended energy at the end of training and cool-down (5 min), with a range total exercise time of 35 to 45 min (Fig. 2). Exercise will be performed for three sessions per week. During the supervised intervention, we will record HR using an HR monitor (Polar Pacer, Lake Success, NY, USA) to ensure compliance with the exercise stimulus at the predetermined target HR zone. In addition, HR and Borg ratings will also be measured in each exercise session.

**Fig. 3:**
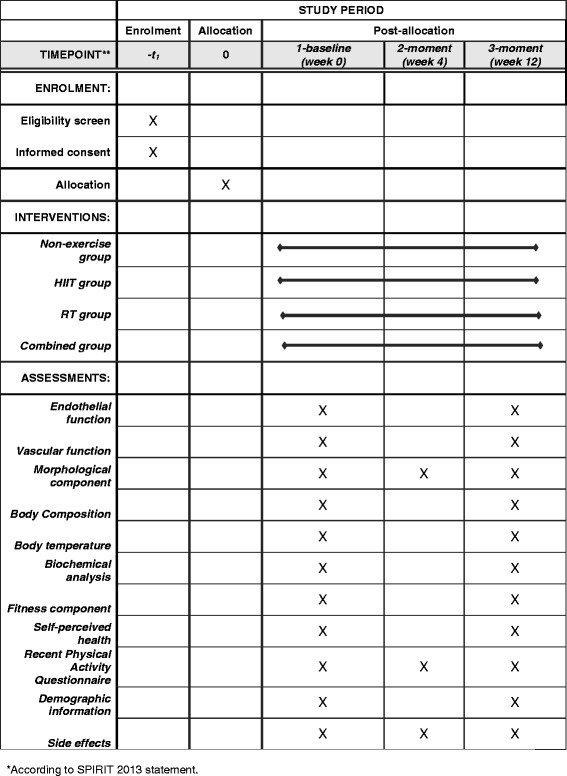
Schedule of enrollment, intervention and assessment*

3. Resistance training group

*Preparatory training phase: weeks 1–4*

All participants will complete a base resistance training protocol during the 2 weeks prior to the training intervention (Table 2). This phase will encompass a total of three workouts (Monday, Wednesday, and Friday) during the 4 weeks. During the adaptation phase, the subjects will expend energy up to 300 kcal at 20–50 % of one repetition maximum (1RM), for 2 × 20–30 repetitions with 1-min rest intervals. During each exercise session, participants will adhere to the 6–8 kcal kg^-1^ week^-1^ energy expenditure format. The purpose of the preparatory phase will be to instruct proper lifting technique, familiarise participants with all exercises, and ensure that the participants began the study with a comparable training base.

**Table 2:** Resistance training programme

Programme variable	Preparatory phase	Volume	Intensity
Preparatory training phase			
Exercise prescription	20–40 % 1RM	40 % 1RM	30–50 % 1RM
Training intensity	2 × 20–30 repetitions	2 × 20–30 repetitions	2 × 20–30 repetitions
Training volume	1 min	30 s	1 min
Energy expenditure	300 kcal		
Rest time	Weeks 1 to 4		
Specific exercises			
Barbell squats			
Split squats			
Biceps curls			
Dumbbell lateral raises shoulder			
Dumbbell military shoulder press			
Dumbbell triceps curls			
Resistance training protocol			
Exercise prescription	40–60 % 1RM	70 % 1RM	70–80 % 1RM
Training intensity	4 × 20–30 repetitions	4 × 20–30 repetitions	4 × 20–30 repetitions
Training volume	1 min	30 s	1 min
Energy expenditure	500 kcal		
Rest time	Weeks 1 to 4		
Specific exercises			
Barbell squats			
Dumbbell squats adductor			
Split squats			
Lateral adductor squat			
Biceps curls			
Dumbbell lateral raises shoulder			
Dumbbell military shoulder press			
Dumbbell triceps curls			

Volume = sets × repetitions. *1RM* one repetition maximum

*RT protocol: weeks 5–12*

After the preparatory phase, participants will expend 500 kcal of energy during the protocol training phase at 40–80 % of 1RM, for 4 × 20–30 repetitions and 1-min rest intervals. The RT protocol will be used to complete a resistance circuit (including upper and lower muscle groups; eight exercises); all participants will expend energy up to the 500-kcal goal. The entire workout will last approximately 30–40 minutes, depending on the number of exercises (Table 2), at a frequency of three times per week. Each session is preceded and followed by a gradual warm-up and cool-down period (both of 10-min duration and consisting of walking and light, static stretching (avoiding muscle pain) in most muscle groups). The cool-down period also includes relaxation and stretching exercises. The RT protocol will be performed through the full range of motion normally associated with correct technique for each exercise, engaging the major muscle groups (abdominal, dorsal, shoulder, upper and lower limb muscles). As a general rule and to avoid potential risks, we will avoid (1) activities that include Valsalva’s mechanism, (2) ballistic and plyometric movements, and (3) positions of extreme muscular tension. This resistance training programme has been used before, successfully promoting strength and muscle gains in middle-aged and elderly populations with a variety of conditions (e.g. diabetes type II and obesity) [33, 34].

4. Combined training group

This group will receive both the HIIT and RT protocols as described above. Therefore, the energy expenditure associated with the physical training prescribed for the vigorous-intensity group will be approximately 1500 kcal/week.

Overall, we will monitor each subject’s HR (FS1, Polar Electro Öy, Kempele, Finland) during the exercise sessions. We will estimate the energy expenditure during the exercise sessions by calibrating the energy expenditure to the HR during the VO_2_max tests performed at the baseline and post-intervention time points. The regression of the energy expenditure will be calculated for each participant according to HR and minutes spent exercising during the training sessions. Trainers will be physical therapists and physical educators with experience developing and monitoring exercise programmes among clinical populations. All protocol training will be performed under observation and supervision in an exercise laboratory with complete and strict monitoring of the amount of exercise completed in each session. Adherence to the exercise programme will be encouraged by the exercise professional who supervises each of the group sessions. To maximise adherence to the training programme each session will have a maximum of three to five participants training simultaneously. Attendance at supervised sessions includes compliance with target HR and expenditure energy and will be monitored and recorded by research staff. Each participant will meet with the study dietician for nutrition assessment and counselling, and an individualised nutrition intervention plan will be developed from the baseline food intake assessment according to participant preferences [35]. This plan is a standardised meal consisting of 1300 to 1500 kcal (50–55 % carbohydrates, 30–35 % total fat, less than 7 % saturated fat and 15–22 % protein).

At the beginning of the training protocol, we will obtain the participants’ weight to determine weekly energy expenditure necessary to achieve their target of 12 kcal kg^-1^ week^-1^ (iso-energetic). It is expected that the gradual increase in total energy expenditure will minimise fatigue, soreness, injuries, and attrition.

### Data collection and outcome measures

Outcome measures will be assessed at baseline and at 12-week follow-up by personnel blinded to the treatment allocation. Data will be recorded on standardised forms and entered into a secured-access database that contains quality control checks (e.g. range checks, notification of missing data).

The primary outcome measure is endothelial function as measured by flow-mediated dilatation (FMD). The secondary outcome variables include HR variability, pulse wave velocity (PWV), weight, BMI, WC, body composition, body temperature, biochemical profile, health-related physical fitness, self-perceived health, health-related quality of life (HRQL), and side effects. Other variables of interest include 24-hour dietary recall, lifestyle and demographic characteristics (Table 3 and Figure 3).

**Table 3:** Summary of time point measurements of the primary and secondary outcomes of the study

Type of outcomes	Specific outcomes	Measurements		
		1-baseline (week 0)	2-moment (week 4)	3-moment (week 12)
Primary outcomes	Endothelial function as measured by flow-mediated vasodilatation	X		X
Secondary outcomes	Vascular function: heart rate variability, aortic pulse wave velocity, augmentation index and blood pressure	X		X
	Morphological component: weight, height, BMI, waist circumference	X	X	
	Body composition: fat mass and non-bone lean body mass	X		X
	Body temperature	X		X
	Plasma samples and biochemical analysis: LDL cholesterol (LDL-c), HDL cholesterol (HDL-c), total cholesterol, triglycerides, glucose, glycated haemoglobin (HbA1c)	X		X
	Fitness component: self-reported fitness, 1RM, hand-grip and peak uptake of volume of oxygen	X		X
	Self-perceived health: health-related quality of life	X		X
	Recent Physical Activity Questionnaire	X	X	X
	Demographic information	X		
	Side effects	X	X	X

*1RM* one repetition maximum, *BMI* body mass index

#### Primary outcome measures

##### Endothelial function

FMD will be measured with the technique introduced by Ramírez-Vélez et al. [36] in the Colombian population using the guidelines reported by Corretti et al. [37]. The diameter of the brachial artery will be assessed using a high-resolution ultrasound device (Acuson Sequoia C512, Acuson Siemens, Mountain View, CA, USA), equipped with a 7.5–10-MHz linear array transducer and an integrated electrocardiography package. The ultrasound procedures will be performed with the subject resting quietly in a supine position for at least 10 min. Measurements will be performed in the morning (06.00–08.00 hours) due to diurnal variation in a climate-controlled room (18–20 °C) with the lights dimmed. All measurements will be taken at the end of diastole as observed by electrocardiogram. First, the diameter of the right brachial artery will be searched in a cross-sectional view and then scanned over a longitudinal section 5 to 10 cm proximal to the right elbow. The diameter of the brachial artery will be measured from the anterior to the posterior intima-lumen interface at a fixed distance, calculating the mean diameter from four cardiac cycles. After this, a pneumatic tourniquet placed around the right forearm will be rapidly inflated to at least 50 mmHg above the systolic blood pressure for 5 min. A sudden release of the cuff will induce an increase in blood flow in the brachial artery located proximal to the tourniquet. During reactive hyperaemia, shear stress will increase, causing endothelium-dependent vasodilatation, mainly due to endothelial release of nitric oxide [38, 39]. This secondary dilatation enhances and prolongs the reactive hyperaemic phase. FMD of the brachial artery will be measured 45–60 s after cuff release. The change in diameter caused by the increased flow will be calculated as the percentage change relative to the baseline measurement (FMD%). The intra-session coefficients of variation will be up to 1 % for baseline diameter. Reliability, estimated by intra-class correlation coefficients (ICC) based on four baseline measurements (*n* = 8 subjects), showed an ICC of 0.91 for the baseline diameter and 0.83 for FMD (*own date*). Images will be recorded on a DVD player for subsequent measurements by one observer blinded to the study.

#### Secondary outcomes

##### Heart rate variability

Measurement of HR variability will be performed according to current recommendations by the European Society of Cardiology using an evaluated share-ware (Kubios, vers. 2.0; http://kubios.uef.fi/) [40]. The variations in interbeat intervals in the time domain will be quantified by the mean values and standard deviations of normal interbeat intervals in the supine position.

##### Aortic pulse wave velocity (PWV) and augmentation index (AIx)

Both PWV and AIx will be measured with the oscillometric method using the occlusion technique. Patient data and the measured distance between the jugulum and the symphysis will be registered in the arteriography-programmed computer (TensioMed Software v.1.9.9.2; TensioMed, Budapest, Hungary). A tape measure will be used for measuring the distance between the jugulum and the symphysis, namely the aortic distance. The cuff will be placed on the patient’s upper arm and connected to the device. Pressure variations in the cuff will influence a pressure receptor and the signal, and will then be transferred via an infrared port to the computer. The algorithm measuring blood pressure in the arteriography device has been validated [41]. PWV will be calculated as the jugulum and the symphysis distance (m) divided by return time (return time/2) (s). For PWV, two recordings with the lowest standard deviation will be chosen. The standard deviation will be calculated from every heartbeat during a period of 8 s. Both aortic AIx (AIxao) and brachial AIx (AIxbr) will be calculated as:

$$ 100\times \left(\mathrm{early}\ \mathrm{systolic}\ \mathrm{pressure}\ \mathrm{peak}-\mathrm{late}\ \mathrm{systolic}\ \mathrm{peak}\right)/\left.\mathrm{pulse}\ \mathrm{pressure}\right). $$

The return time is the difference (in ms) between the first (early systolic pressure peak) and reflected systolic wave (late systolic peak) and is related to the stiffness of the aorta. The PWV and AIx will be presented as the mean values from two recordings. The algorithm for estimation of central systolic blood pressure (cSBP) has been derived from the relationship between invasively measured cSBP and the SBP in the brachial artery, and cSBP estimated by arteriography correlates well with invasively measured cSBP [41]. Blood pressure will be measured at the same time of the day using a validated digital automatic blood pressure monitor (OMRON M6, Omron Health Care Co., Ltd., Kyoto, Japan) according to the International Protocol of the European Society of Hypertension [42].

##### Morphological component

Anthropometric variables will be assessed by a nutritionist in accordance to the International Society for the Advancement of Kinanthropometry (ISAK) guidelines [43]. Variables will be collected at the same time in the morning, between 00.70 and 00.80 hours, following an overnight fast of at least 10–12 hours. The body weight of the subjects will be measured when the subjects are in underwear and without shoes, using electronic scales (Tanita® BC544, Tokyo, Japan). The height of the subjects will be measured using a mechanical stadiometer platform (Seca® 274, Hamburg, Germany). The BMI of the subjects will be calculated as the body weight in kilograms divided by the square of the height in metres. The WC will be measured as the narrowest point between the lower costal border and the iliac crest; in case this is not evident, it will be measured at the midpoint between the last rib and the iliac crest using a tape measure (Ohaus® 8004-MA, Parsippany, NJ, USA). The waist-to-height ratio will be computed by dividing WC by height, and this provides a surrogate measure of central body fat. We will take each measure twice and use the average measure obtained, unless the first and second measures vary by more than 1 %, in which case we will use the median of three measurements.

##### Body composition

We will also measure fat mass, lean body mass, abdominal adipose tissue and bone mineral density by conducting a dual-energy X-ray absorptiometry scan (DEXA) (Hologic, QDR 4500 W). An experienced DEXA technologist who is blinded to the study randomisation will perform the DEXA imaging studies.

##### Body temperature

We will use a thermo-infrared camera (FLIR Thermacam E60, FLIR systems, Boston, MA, USA). Images in the frontal plane will be taken from the anterior and posterior sides, according to the thermal image acquisition criteria described by Ring and Ammer [44]. During the measurement process, participants will remain in their underwear and will maintain a steady orthostatic position during the image acquisitions. The distance from the camera to the subjects will be 2.5 m. The study will be conducted according to the guidelines of the American Academy of Thermography [45]. A single investigator, trained in the use of thermographic devices, will obtain the images.

##### Plasma samples and biochemical analysis

A fasting blood sample will be obtained from the cubital vein in the early morning at the clinical care session attended by the participants in the subset. The biochemical profile will include: (1) plasma lipid triglycerides, total cholesterol, high-density lipoprotein cholesterol (HDL-c), low-density lipoprotein cholesterol (LDL-c) (by enzymatic colourimetric methods); and (2) the metabolic regulators glucose and haemoglobin A1C (HbA1c) (by enzymatic colourimetric methods). All determinations will be analysed in serum using a Cardiocheck® and A1CNow^+^® system.

##### Self-reported fitness

Self-reported fitness will be determined by the International Fitness Scale (IFIS), which is a questionnaire validated in European [46] and Colombian adults [47]. IFIS consists of a Likert-type scale (range 1–5) with five response options (very poor, poor, average, good, and very good) about perceived overall fitness; its main components include CRF, muscular strength, speed and agility, and flexibility (http://www.helenastudy.com/IFIS). IFIS has shown ‘high’ validity and ‘moderate’ to ‘good’ reliability in young adults [48]. In Colombian adults, the internal consistency and reproducibility of IFIS items was high (Cronbach’s alpha = 0.80) and the averaged ICC range was 0.90–0.96 [47].

##### Health-related physical fitness

Physical fitness will be measured using tests that have previously shown high validity and reliability levels. It will be determined using a maximum treadmill exercise test (Precor TRM 885, Treveso, Italy) following the modified Balke protocol [49], which has been extensively used [50, 51] and validated [52]. The treadmill test will use a ramp protocol where the inclination is constant (5.5 %) and the speed increased by 0.5 km/h every minute, starting at 4 km/h. Each session will begin with a 5- to 10-min warm-up at 50 W. We will ask participants to refrain from smoking for 2 hours before the test, and from drinking alcohol or doing any vigorous- or moderate-intensity activities for 48 h before the test. HRmax will be used to determine the training intensity for each participant. We will measure blood pressure prior to and during the test. Exercise will be terminated if participants are fatigued, or earlier if they fulfill the ACSM’s guidelines for ‘Indications for Terminating Exercise Testing’ [53]*.* CRF is defined as the highest recorded VO_2_ value (VO_2_max) after two of three criteria are met: (1) a plateau in VO_2_ after increase in workload, (2) a respiratory exchange ratio >1.10, and (3) a maximal HR within 10 bpm of their age-predicted maximum. Muscular fitness will be assessed using the hand-grip test (maximum hand-grip strength assessment) using a standard adjustable handle analogue hand-grip dynamometer T-18 TKK SMEDLY III® (Takei Scientific Instruments Co., Ltd, Niigata, Japan). Participants will be given a brief demonstration and verbal instructions for the test and, if necessary, the dynamometer will be adjusted according to the subject’s hand size according to predetermined protocols. Hand-grip strength will be assessed with the subject in a standing position with their shoulders adducted and neutrally rotated, and their arms parallel but not in contact with their body. The participants will be asked to squeeze the handle for a maximum of 3–5 s, but no verbal encouragement will be given during the test. Two trials will be allowed in each limb, and the average score will be recorded as peak hand-grip strength (kg). Thus, the values of hand-grip strength presented will combine the results of left- and right-handed subjects, without consideration of hand dominance. Hand-grip will be adjusted by allometric parameters defined by Jaric [54] (dynamometry/weight^0.67^). Strength in the eight exercises will be assessed at baseline and immediately after the intervention ends. A general warm-up consisting of riding a cycle ergometer for 5 min at a self-selected resistance will precede strength testing. Standardised procedures will be used to predict a one-rep max (1RM) from reps-to-fatigue barbell squats, dumbbell adductor squats, split squats, lateral adductor squat, biceps curls, dumbbell lateral shoulder raises, dumbbell military shoulder press, and dumbbell triceps curls from each participant’s performance during the preparatory training phase [55]. Progressive overload will be achieved by increasing the load when all prescribed repetitions (for a particular exercise) are achieved on two consecutive workouts [56].

##### Recent Physical Activity Questionnaire (RPAQ)

Self-reported physical activity is measured using the RPAQ. This assesses physical activity across four domains (domestic, recreational, work, commuting) over the previous month. It has shown moderate-to-high reliability for physical activity energy expenditure and good validity for ranking individuals according to their time spent in vigorous intensity physical activity and overall physical activity energy expenditure [57].

##### Health-related quality of life (HRQL)

HRQL will be measured by the SF Community - short-form survey (SF*-*12™) Colombian version for physical and mental domains’ summary scores and eight subscales (including vitality) [58]. The internal consistency of the HRQL items is moderate (Cronbach’s alpha = 0.70).

##### Sociodemographic information

Baseline sociodemographic values, which could act as covariates or confounds for the tested treatment modality, will also be collected. The surveys will include questions on age, education, occupation, income, health history, and alcohol consumption, among others.

##### Dietary assessment

To determine the average habitual energy and macronutrient intake, a detailed 24-h diet record will be obtained from all subjects 1 weekday and 1 weekend day during the 1-week baseline period. The Food Intake Analysis Software (FAO/INFOODS, Report of the Technical Workshop on Standards for Food Composition Data Interchange, Rome 2004) and national food composition tables (for specific foods) will be used to analyse total energy and macronutrient intake of each subject’s 24-h diet.

##### Side effects and monitoring

The study will be conducted according to good clinical practice and standard operating procedures. It will be monitored by the Human Rights Committee at the Universidad Manuela Beltrán Coordinating Centre composed of experts in physical exercise, sports medicine, physical therapists, physical educators and clinical epidemiologists. Interim monitoring reports will be submitted to the experts, focusing on patient intake, adherence to the protocol, baseline comparability of treatment groups, completeness of data retrieval, and adverse events. All adverse events will also be reported to the Universidad Manuela Beltrán Ethics Committee. To standardise the study procedures, an operations manual has been written, and comprehensive training sessions will be held prior to the initiation of the trial. An independent researcher will be in charge of auditing all assessment staff to record all of these events for the participants over the study period.

### Power calculation and sample size

The aim of this study is to obtain data on the effects of HIIT, RT or combined training that will result in similar improvements in cardiometabolic health compared to the usual clinical care group. The measurement of FMD, validated in several population studies, was selected as the critical variable to calculate the sample size [59, 60]. To calculate the required sample size, we will use the formula for the comparison of two means:$n={\left[A+B\right]}^2\times 2\times S{D}^2/DIF{F}^2,$ where *n* = the sample size required in each group, *SD* = standard deviation of the outcome variable, and *DIFF* = size of desired difference between groups. *A* and *B* depend on the desired significance level and desired power, respectively. Using estimates obtained from the literature [59, 60] and our previously performed study [61, 62], a sample size of 12 subjects in each group will be needed to reach a power of 80 % to detect a difference in means in the FMD of 2 % in the FMD after 12 weeks of training, assuming a SD of 2.7 using a two-sample *t* test with a 0.05 two-sided significance level. Assuming a drop-out rate of 15 %, the total minimal sample size has been increased to 15 subjects for each group. We believe that this sample size is feasible and realistic based on our previous experiences in RCTs [33, 34, 59, 60, 63, 64].

#### Statistical analyses

The final data will be analysed using IBM SPSS 22.0 (SPSS, Inc., Chicago, IL, USA) and SAS software (SAS Institute Inc., Cary, NC, USA). An exploratory analysis will be performed to determine the frequency, range, variability, and distribution type for each variable to use the most appropriate statistical test when comparisons will be necessary. These analyses permit the assessment of the primary analysis of the data and will be undertaken using the principle of intention-to-treat (ITT). The ITT analysis for this study will include all participants, including those who are not fully compliant and those with missing outcome data. Because this is an experimental design with two measures of the primary and secondary outcomes, the first at baseline x0 (t0 = weeks) and the second after interventions x1 (t1 = 12 weeks) in four study groups, a comparative analysis between these measures to establish differences will be executed. To perform these comparisons, one-way ANOVA or Kruskal-Wallis tests will be applied when appropriate. Subsequently, a multivariate analysis will be carried out, and the autocorrelation between repeated measures will be taken into account. We will use longitudinal analysis methods, such as a generalised estimating equation approach, to control the differences among measures at baseline and to incorporate incomplete observations into this analysis. Finally, we will investigate if there is an interaction between the two interventions for the primary outcome at 12 weeks of supervised training HIIT, RT or combined training (HIIT + RT). For these analyses, we will include appropriate interaction terms in the models [38, 65]. The trial is not powered to detect these interactions and is likely to have poor precision for the interaction terms. We plan to report regression coefficients for the interaction terms and their 95 % confidence intervals. However, we aim to recruit 15 participants per group (a total of 60) to accommodate for a 20 % attrition rate and elimination due to non-compliance.

## Dissemination

We will disseminate the results of our study via presentations at international conferences and publications in peer-reviewed journals. The study will be implemented and reported in line with Standard Protocol Items for Randomised Trials [66] Additional file 1.

## Discussion

This protocol describes a study in which we will compare the effects of 12 weeks of HIIT, RT or combined training on improvements in body composition, endothelial function, blood pressure, blood lipids, and cardiovascular fitness in a cohort of sedentary, overweight adults (aged 30–50 years).

Latin American countries are experiencing different stages of nutrition transition. Although the prevalence of undernutrition is declining at different rates, the prevalence of overweight is dramatically increasing [67]. In addition, physical inactivity and sedentary lifestyles are also a preventable behaviour associated with obesity, but the evidence on this issue remains mixed [68], and the results might not be generalisable to all world regions [69]. Thus, determining whether HIIT + RT can be a viable public health approach to improve cardiometabolic health is warranted, particularly given a recent finding that HIIT added to plyometric exercise is more consistently associated with improvements in metabolic abnormalities than HIIT alone.

We believe that our trial could help to determine which type of programme better improves the cardiometabolic health of overweight adults and makes more efficient use of an adult’s resources over time. In summary, the primary objective of the trial is to contribute to the growing body of literature about physical exercise interventions and cardiometabolic health among sedentary, overweight adults living in Bogotá, which will be useful for designing innovative and time-efficient preventive measures among the Colombian population.

## Trial status

The Cardiometabolic HIIT-RT Study began recruiting patients in March 2016 and will close recruitment in October 2016. Data collection will be completed in June 2017.

## Abbreviations

AIx, augmentation index; AIxao, aortic augmentation index; AIxbr, brachial augmentation index; BMI, body mass index; CRF, cardiorespiratory fitness; cSBP, central systolic blood pressure; CVD, cardiovascular diseases; DEXA, dual-energy x-ray absorptiometry scan; FMD, flow-mediated dilatation; HbA1c, haemoglobin A1C; HDL-c, high-density lipoprotein cholesterol; HIIT, high-intensity interval training; HRmax, maximum heart rate; HRQL, health-related quality of life; IFIS, International Fitness Scale; ISAK, International Society for the Advancement of Kinanthropometry; LDL-c, low-density lipoprotein cholesterol; MET, metabolic equivalents; MICT, moderate-intensity continuous training; PWV, aortic pulse wave velocity; PWV, pulse wave velocity; RPAQ, Recent Physical Activity Questionnaire; RT, resistance training; SF-12™ SF Community - short-form survey; VO_2_max, maximal oxygen uptake; WC, waist circumference

## Additional file

Additional file 1: SPIRIT 2013 Checklist: recommended items to address in a clinical trial protocol and related documents. (DOC 122 kb)
